# Caterpillar-inspired soft crawling robot with distributed programmable thermal actuation

**DOI:** 10.1126/sciadv.adf8014

**Published:** 2023-03-22

**Authors:** Shuang Wu, Yaoye Hong, Yao Zhao, Jie Yin, Yong Zhu

**Affiliations:** ^1^ Department of Mechanical and Aerospace Engineering, North Carolina State University, Raleigh, NC 27695, USA.; ^2^ Department of Materials Science and Engineering, North Carolina State University, Raleigh, NC 27695, USA.; ^3^ Joint Department of Biomedical Engineering, University of North Carolina-Chapel Hill and NC State University, Chapel Hill, NC 27599, USA.

## Abstract

Many inspirations for soft robotics are from the natural world, such as octopuses, snakes, and caterpillars. Here, we report a caterpillar-inspired, energy-efficient crawling robot with multiple crawling modes, enabled by joule heating of a patterned soft heater consisting of silver nanowire networks in a liquid crystal elastomer (LCE)–based thermal bimorph actuator. With patterned and distributed heaters and programmable heating, different temperature and hence curvature distribution along the body of the robot are achieved, enabling bidirectional locomotion as a result of the friction competition between the front and rear end with the ground. The thermal bimorph behavior is studied to predict and optimize the local curvature of the robot under thermal stimuli. The bidirectional actuation modes with the crawling speeds are investigated. The capability of passing through obstacles with limited spacing are demonstrated. The strategy of distributed and programmable heating and actuation with thermal responsive materials offers unprecedented capabilities for smart and multifunctional soft robots.

## Teaser

Distributed programmable thermal actuation enables caterpillar-inspired bidirectional locomotion for soft crawling robots.

## INTRODUCTION

Soft robots have attracted wide attention in biomedical engineering, surgical assistance, active prosthetics, camouflage, and perception technologies. Lots of inspiration have been taken from the animal world to incorporate soft materials with mechanical design for soft robotics, e.g., octopuses (*1*, *2*), fish (*3*, *4*), snakes (*5*, *6*), worms (*7*, *8*), and caterpillars (*9*, *10*). Some unique features of these animals, including multimodal locomotion and passing through confined gaps, can be beneficial in complex and unstructured environments.

Researchers have also been exploring different actuation methods for soft robots using a variety of stimuli, including pressure (*2*, *11*, *12*), heat (*13*–*17*), electrical field (*3*, *18*, *19*), magnetic field (*20*–*22*), and chemical potential (*23*, *24*). Among the various types of stimuli, electric stimulus is one of the simplest and most convenient, where electroactive polymers, either ionic or field activated, are widely used. For electrically stimulated actuators, the ionic activation typically operates in an electrolyte environment, while the field activation requires high voltage (>1 kV) (*25*). Another type of electrically stimulated actuator, thermal bimorph actuator (*13*, *26*, *27*), based on mismatch in coefficient of thermal expansion (CTE) of two materials has drawn much attention due to programmable operation (*28*), lightweight, low actuation voltage, being electrolyte-free, and potential for untethered operation (e.g., via wireless charging) (*29*, *30*).

Among different thermal responsive materials, liquid crystal elastomer (LCE), a thermally driven actuating material that combines polymer network and liquid crystal mesogens, has recently attracted much attention because of its unique properties, including large (~40%) and reversible actuation, high processability, and programmability. As the temperature increases, liquid crystal mesogens transition from the nematic phase to the isotropic phase, leading to a notable and macroscopic deformation in the material. A variety of LCE-based actuators have been designed and fabricated, which are often actuated by direct environmental heating (*31*, *32*), photothermal effects (*33*–*36*), and electrothermal actuation (*37*–*41*). However, for most practical applications, electronically powered actuators provide notable convenience for system control and integration. Some recent studies have successfully integrated stretchable resistive heaters with LCE for better control with electrical signals (*37*, *39*).

Here, we present a caterpillar-inspired bidirectional crawling robot with multiple locomotion modes, enabled by joule heating of distributed programmable silver nanowire (AgNW) heaters in an LCE-based thermal bimorph actuator. With the designed AgNW heating pattern and programmable heating, different temperature distributions and curvature distributions were achieved, resulting in different friction competition between the front and rear ends with the ground and hence bidirectional locomotion. To demonstrate the function of the crawling robot in potential applications, we characterized the performances in forward and reverse locomotion and tested a scenario of passing through a confined gap. The locomotion modes, the crawling speed, and the ability of passing through obstacles with small gap have been studied with experiment and finite element analysis (FEA).

## RESULTS

**Fig. 1.**
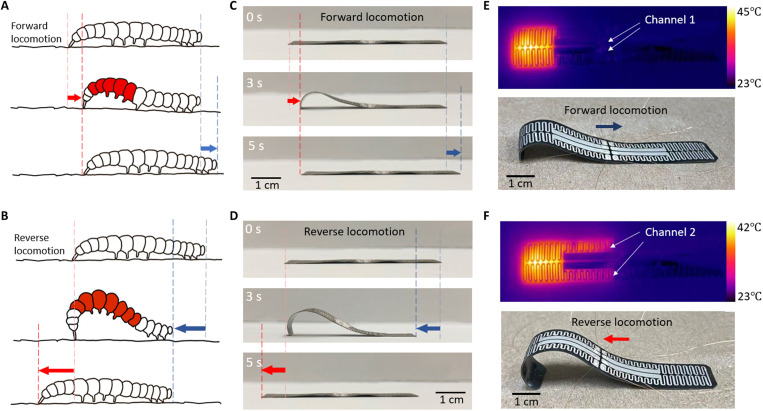
Bioinspired crawling motions. (**A**) Schematics of the forward locomotion of a caterpillar. (**B**) Schematics of the reverse locomotion of a caterpillar. (**C**) Snapshots of the crawling robot in one cycle of actuation for reverse locomotion. (**D**) Snapshots of the crawling robot in one cycle of actuation for forward locomotion. (**E**) infrared image of the crawling robot with 0.05-A current injected in channel 1 and the tilted view of the crawling robot. (**F**) Infrared image of the crawling robot with 30-mA current injected in channel 2 and the corresponding tilted view of the crawling robot.

In nature, the mother-of-pearl moth, *Pleurotya ruralis*, exhibits two-directional locomotion (*42*, *43*). During the forward locomotion, the caterpillar contracts a few hind segments while anchoring the front to move the tail forward, producing a characteristic traveling hump on the back. Subsequently, it releases the hump while anchoring the terminal tip. The entire caterpillar becomes flat again and as a result moves one step forward (Fig. 1A). During the reverse locomotion, the caterpillar anchors the terminal tip on the ground followed by a powerful contraction of the middle part of the body. This motion produces a large hump that arches up the whole body. Then, while anchoring the front part, the caterpillar releases the hump, becoming flat again and moving one step backward (Fig. 1B). The key feature enabling the two-directional movement here is the control of the body curvature. While the caterpillar kinematics involve more sophisticated active control of the body parts at different segments, a caterpillar-inspired crawling robot that can control the local curvature of the body could mimic the same two-directional movement. Figure 1 (C and D) shows the forward and reverse locomotion of a crawling robot, when different heating channels (or patterns) are heated. When the heater is turned off, the relaxation of the bent bimorph structure brings the actuator either forward or backward to finish one cycle of locomotion. Figure 1 (E and F) shows the corresponding infrared images and tilted views of the actuator when channels 1 and 2 are heated, respectively. In Fig. 1 (C and D), a constant current applied to the inner two electrodes (channel 1) and outer two electrodes (channel 2) leads to the forward and reverse locomotion, respectively.

**Fig. 2.**
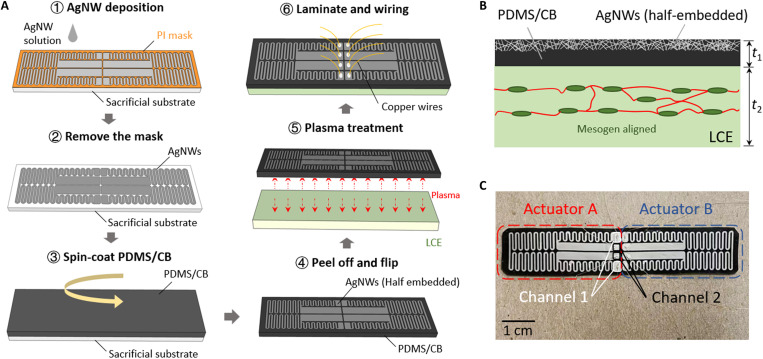
Design and fabrication of the caterpillar inspired crawling robot. (**A**) Fabrication steps of the caterpillar inspired crawling robot. PI, polyimide. (**B**) Cross-section view of the fabricated sample. The AgNWs are half-embedded below the top surface of PDMS/CB composite. The mesogens in the LCE ribbon are aligned through tensile stretching. (**C**) Top view of the crawling robot with symmetric actuator A and actuator B. Each actuator contains two conductive channels (1 and 2).

Figure 2A shows the fabrication process of the crawling robot. AgNWs have been widely used as a heating material in soft devices due to their excellent electric conductivity and mechanical compliance (*13*, *44*–*46*). In this work, we used AgNWs as the heating element, embedded just below the surface of a polydimethylsiloxane (PDMS) matrix (*47*–*49*). The crawling robot is a bimorph structure with a AgNW/PDMS and carbon black (CB) composite film laminated on top of an LCE ribbon. The AgNW pattern was defined by drop casting AgNW solution on top of a masked Si substrate; the AgNWs were in the form of a percolation network structure (see fig. S1). CB powders were doped inside the PDMS precursor to enhance thermal conductivity. Then, the liquid PDMS/CB composite was dropped on top of the AgNW network and cured. The AgNW network was half-embedded below the surface of the PDMS/CB matrix (Fig. 2B). Note that the thermal conductivity of PDMS/CB (with a weight ratio of 4:1) was increased by 31% when compared with pure PDMS but no obvious change of Young’s modulus (<2%). (fig. S2) The LCE ribbon was fabricated by mechanical stretching of a rectangular flat LCE strip synthesized by two-stage polymerization (*50*, *51*). Plasma treatment and mechanical pressure were applied to form strong bonding between the AgNW/PDMS/CB composite film and the LCE ribbon.

When electric current is applied to the AgNW network, heat is generated as a result of Joule heating and transferred to the PDMS/CB composite layer and the LCE layer. Note that the half-embedded AgNW structure is above the PDMS/CB and LCE layers (Fig. 2B). This is because the PDMS/CB surface can form stronger bonding with the LCE surface than the AgNW/PDMS/CB surface. As the AgNW/PDMS/CB layer is relatively thin (60 μm), this configuration does not sacrifice much of the heating efficiency. When the temperature increases, the PDMS/CB composite expands due to thermal expansion, while the LCE layer shrinks due to the nematic-isotropic transition. Figure 2C shows the top view of the crawling robot with two symmetric actuators (A and B). Each actuator contains two conductive channels (1 and 2). By designing the AgNW heater pattern and hence tailoring the temperature distribution, different kinematics of the crawling robot can be achieved.

The performance of the bidirectional locomotion entails three major aspects: the heater performance, the frictional force analysis, and the effect of the amplitude and frequency of the power supply.

**Fig. 3.**
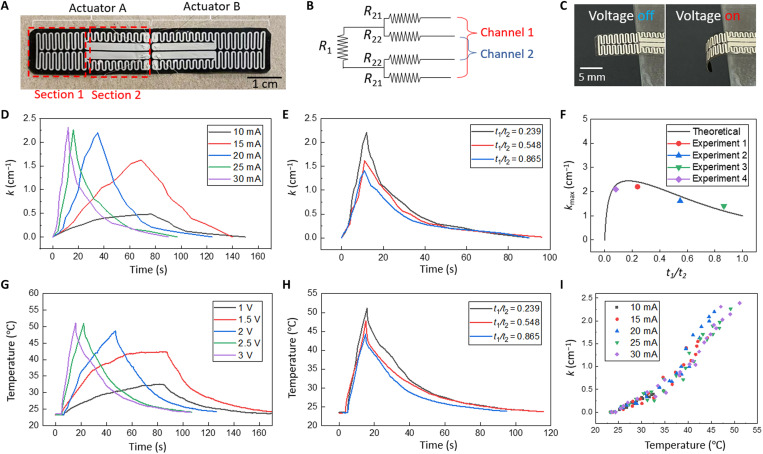
Heating performance of the soft crawling robot. (**A**) Photograph of the heating pattern with two sections on each actuator. (**B**) Diagram of the two-channel electrical circuit corresponding to the heating pattern in (A). (**C**) Photograph of the bimorph before (left image) and after (right image) the heater is turned on. (**D**) Curvature of the bimorph cantilever with respect to time with different current applied. (**E**) Curvature of the bimorph cantilever with respect to time with different thickness ratio between the two layers of the bimorph. (**F**) Theoretical prediction of maximum curvature compared with the experimental results. (**G**) Temperature of the bimorph cantilever with respect to time with different current applied. (**H**) Temperature of the bimorph cantilever with respect to time with different thickness ratio. (**I**) Relationship between the curvature and temperature of the bimorph cantilever.

The conductive AgNW pattern is composed of two symmetric parts, each containing two sections, as shown in Fig. 3A. Section 1 is uniformly covered with a serpentine shaped conductive trace with line width of 0.65 mm. Section 2 is composed of two groups, each containing a thin serpentine trace (0.65 mm in width) and a thick straight line (2.4 mm) in parallel. The equivalent circuit model is shown in Fig. 3B. In this study, the as-fabricated AgNW/PDMS composite has a uniform sheet resistance of 0.5 ohm sq^−1^. The resistance components as shown in Fig. 3B, R_1_, R_21_, and R_22_, are 115.4, 38.8, and 3.2 ohm, respectively. As a result, channel 1 has a resistance of 193 ohm and channel 2 has a resistance of 121.8 ohm.

To characterize the heating and actuation performance of the crawling robot, we conducted a parametric study on the effect of the electrical current and the bilayer thickness ratio between the AgNW/PDMS/CB film and the LCE ribbon (*t*_1_/*t*_2_), with section 1 of the device cantilevered on a fixed boundary (Fig. 3C). Figure 3D shows the curvature of the sample (with constant *t*_1_/*t*_2_ = 0.239) as a function of time under different currents from 10 to 30 mA. With the increasing current, the heating time significantly dropped from 80 to 12 s. Note that the power supply was stopped when the curvature reached the maximum value of 2.3 cm^−1^ (the sample bent into a circle). Figure 3E shows the curvature with respect to time with different thickness ratios (*t*_1_/*t*_2_ = 0.239, 0.548, and 0.865). With the same applied current (25 mA) and the same heating time, the sample with *t*_1_/*t*_2_ = 0.239 yields the largest bending curvature. The curvature of a bimorph can be calculated with the Timoshenko’s equation (*52*)

$$k=\frac{6(\alpha_{1}-\alpha_{2})(T-T_{0}){\left(1+m\right)}^{2}}{h[3{\left(1+m\right)}^{2}+(1+mn)\left(m^{2}+\frac{1}{mn}\right)]}$$ (1)

where $m=\frac{t_{1}}{t_{2}}$ with *t*_1_ and *t*_2_ as the thicknesses of the two layers (AgNW/PDMS/CB layer and LCE layer, respectively), *h* = *t*_1_+*t*_2_, $n=\frac{E_{1}}{E_{2}}$ with *E*_1_ and *E*_2_ as the Young’s moduli of the two layers, *T*_0_ is the initial temperature, *T* is the temperature of the actuator, and α_1_ and α_2_ are the coefficients of thermal expansion of the two layers, respectively. The CTE of PDMS is 3.1 × 10^−3^°C^−1^ (derived from the data sheet of Dow Inc.). The CTE of LCE is −2.24 × 10^−3^°C^−1^ (derived from the measured strain/temperature relationship of fabricated LCE ribbon from room temperature to 45°C). Note that the temperature *T* is from the infrared (IR) measurement on the top of the actuator. There is a small temperature gradient in the thickness direction (~0.3°C) (see fig. S3). Therefore, the theoretically predicted curvature is slightly overestimated. However, the curvature difference caused by such a small temperature difference is negligible. To provide the first-order guide to our design, we used a simplified model with uniform temperature in the thickness direction.

Figure 3F plots the theoretical prediction of the curvature according to Eq. 1 and the experimental results, which agreed well. Note that in Eq. 1, we neglected the Young’s modulus contribution from AgNWs because the AgNW-embedded layer is only 3 μm, while the whole PDMS/CB layer is generally 20 times thicker. The Young’s modulus of LCE was measured by uniaxial tensile testing (fig. S4). Thus, in the following discussion, we choose *t*_1_/*t*_2_ = 0.239 for the bending actuation. Similarly, the temperature with respect to time has been plotted for different current and different thickness ratios (Fig. 3, G and H). The temperature of the bimorph was taken using an IR camera focusing on the top surface of the PDMS/CB composite area. Last, the curvature as a function of the temperature for the samples with *t*_1_/*t*_2_ = 0.239 is plotted in Fig. 3I. A nonlinear relationship can be seen, although Eq. 1 predicts a linear relationship. This can be explained by the nonlinear relationship between α_2_ and the temperature. Figure S5 shows that α_2_ in the LCE layer gradually increases with the increase of temperature from room temperature to 75°C and then starts to decrease until 145°C. As a result, the curvature of the bimorph structure shows a slope increase when the temperature rises within the range shown in Fig. 3I. With increase of the temperature, the Young’s modulus of the LCE also increases but with negligible influence on the curvature based on Eq. 1.

**Fig. 4.**
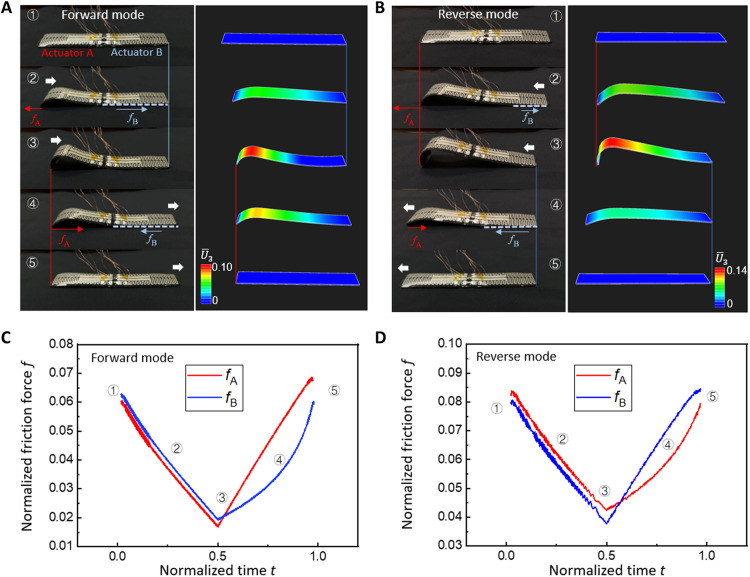
Two crawling modes of the caterpillar robot. (**A**) Comparison between the images and simulation results (color bar representing the normalized out-of-plane deformation) of the robot in forward mode. (**B**) Comparison between the images and simulation results of the robot in reverse mode. (**C** and **D**) Friction force on two ends of the crawling robot in forward mode and reverse mode normalized by the self-weight of the robot.

In Fig. 4A, the left panel shows the snapshots of the forward locomotion mode of the caterpillar robot (movie S1). In snapshot 2, when the channel 1 of actuator A (left half of the robot) is activated (forward mode), actuator A starts to arch and causes a friction competition between the left end and the right end (*f*_A_ and *f*_B_). Because of the asymmetry of the arc shape, *f*_A_ is hypothesized to increase and reach the sliding friction criteria before *f*_B_ does. As a result, the left end slides rightward, while the right end stays stationary. When the power is off, the relaxation of the asymmetric arc shape causes the friction forces *f*_A_ and *f*_B_ to switch direction simultaneously and start a new competition. This time, *f*_B_ reaches the sliding friction first and starts to move rightward, while the left end is anchored until the whole robot returns to the initial flat state. Similarly, the left panel of Fig. 4B shows reverse mode of actuator A when the channel 2 of actuator A is activated (movie S2). In snapshot 2, the asymmetric arch shape shows a very different curvature distribution compared with snapshot 2 in Fig. 4A. In reverse mode, more of the middle part of the robot is lifted up, leaving a smaller contact area between the right end and the ground. Such a difference in contact area with the ground results in an opposite friction competition outcome. Snapshots 2 and 3 show that *f*_B_ reaches the sliding force first. The right end continues to move leftward, while the left end stays anchored. Similarly, when the power is off, relaxation of the arch shape leads to leftward motion of the left end, while the right end stays anchored.

To validate the hypothesis above, we conducted FEA using Complete Abaqus Environment (Abaqus/CAE). The soft crawler is modeled as a bilayer three-dimensional (3D) deformable structure, and the ground is modeled as a rigid surface. A friction coefficient (0.3) is applied between the bottom and side surfaces of the crawler and the substrate. The coefficient of friction is experimentally measured by dragging the deformed crawling robot on the substrate. The defined heating area is the same as observed from the IR images in experiment. The snapshots of the simulated results agree very well with experimental results in terms of locomotion direction and relative out-of-plane displacement ($\bar{U_{3}}$ normalized by the length of the actuator). The friction forces on the two ends of the crawling robot are extracted from the simulation and normalized by the self-weight of the robot (Fig. 4, C and D). Point ② in Fig. 4C (forward mode) shows that *f*_A_ < *f*_B_, which causes the sliding of the left end. However, starting from point ③, *f*_A_ increases more than *f*_B_ and causes the right end of the crawling robot to slide on the ground. In reverse mode (Fig. 4D), *f*_A_ > *f*_B_ in the first half of actuation cycle. When the power is off, *f*_A_ drops below *f*_B_ for the rest of the actuation cycle. Hence, reverse mode shows a completely opposite direction of locomotion compared with forward mode. The opposite sliding sequence of actuator A and actuator B is a result of different centroid location and touching angle with the ground when the robot bends to a different shape. The simulation results validated the friction competition mechanism of the crawling robot when distributed heating is applied.

**Fig. 5.**
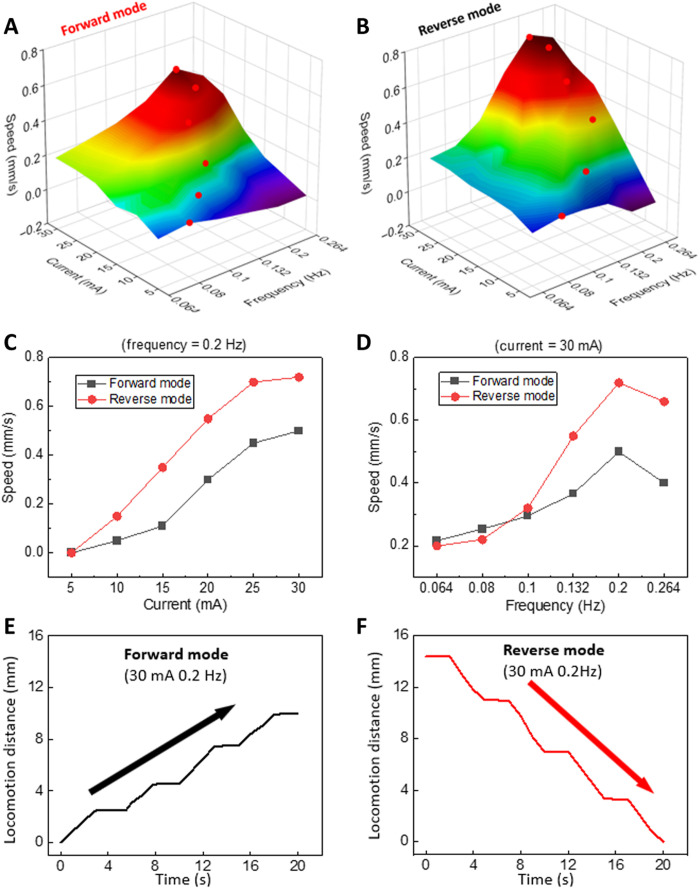
Locomotion speed of the crawling robot. (**A**) Forward mode and (**B**) reverse mode with different current and actuation frequency. (**C**) Locomotion speed of forward and reverse mode with different current at a constant actuation frequency of 0.2 Hz. (**D**) Locomotion speed of the forward and reverse mode with different frequency at a constant current of 30 mA. (**E** and **F**) Locomotion displacement (at 30 mA and 0.2 Hz) of the crawling robot in forward and reverse mode, respectively.

Figure 5 shows the locomotion speed (in forward mode and reverse mode) of the crawling robot as a function of the applied current (from 5 to 30 mA) and actuation frequency (from 0.064 to 0.264 Hz). In general, both forward and reverse mode show increasing speed when the applied current increases (Fig. 5, A and B). It can be observed more clearly in the speed versus current plot as shown in Fig. 5C. However, in terms of speed versus frequency (Fig. 5D), the locomotion speed first increases with the increasing actuation frequency but then decreases. When it reaches the maximum value (highlighted in red dots in Fig. 5, A and B), further increase of the frequency decreases the locomotion speed. This is due to the minimum heating and cooling time required during each actuation cycle. It is straightforward that when the actuation frequency is low, the locomotion speed is low. However, when the frequency is too high, within each cycle, the time for cooling is too short for the crawling robot to become flat again or even the time for heating is not sufficient to reach the target curvature. As a result, the locomotion speed drops. Compared with the forward mode, reverse mode is generally faster. As shown in Fig. 5 (E and F), under 30 mA and 0.2 Hz, the speed of the forward and reverse locomotion is 0.5 and 0.72 mm/s (0.008 and 0.012 body length/s), respectively. This is because in reverse mode, a larger part of the robot contracts and generates a longer stride for each crawling step, consistent with the caterpillar locomotion. The actuation speed of thermal actuators can be further improved with faster thermal response (e.g., reducing the LCE thickness and increasing its thermal conductivity) or introducing some mechanical designs, e.g., the instability design (*17*). For the purpose of comparison, the reported speed of the forward and reverse locomotion in (*39*) using the same electrothermal actuation mechanism and LCE material is 0.032 and 0.021 mm/s (0.0011 and 0.0008 body length/s), respectively.

**Fig. 6.**
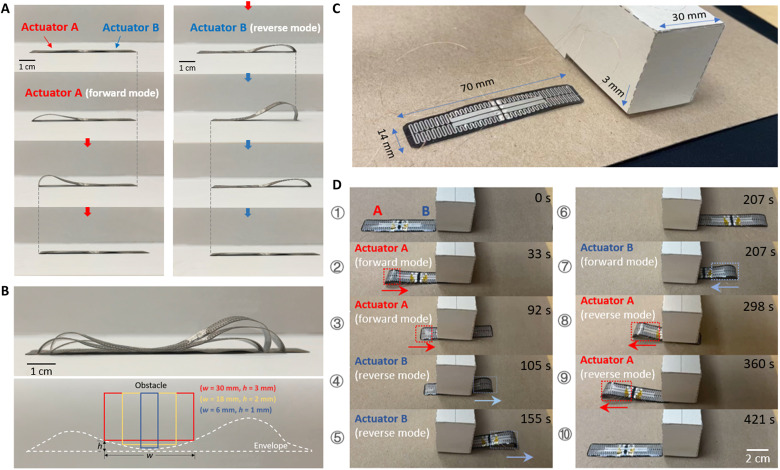
Demonstration of the crawling robot passing through a shallow deep gap. (**A**) Side view of the crawling robot during the transition from actuator A to actuator B. (**B**) Overlapped photographs showing the history of motion in (A) and corresponding schematic showing the obstacles that the crawling can pass through. (**C**) Comparison between the crawling robot and the obstacle, which forms a confined tunnel with the floor. (**D**) Snapshots of the robot passing through this confined tunnel and reversely passing again to return to the initial location.

Last, because of the symmetric two-actuator design (e.g., Fig. 2C) in our crawling robot and the two-directional locomotion capability for each actuator, we demonstrated the application of the soft crawling robot by passing through small, confined space with a much lower gap height than that of the robot (movie S3). Figure 6A shows the side view of the crawling robot in motion—it starts with actuator A with a current applied to channel 2 for one cycle and then switches to actuator B with a current applied to channel 1 for another cycle. Because of the symmetricity of the two actuators, this actuator transition together with mode transition does not change the moving direction for the whole device. By overlapping all the snapshots during this entire motion, we can observe an envelope contour (dashed line in Fig. 6B) that reveals a deep valley in the middle. This interesting body profile can facilitate the crawling robot to pass under obstacles with confined space. The schematic in Fig. 6B shows a few examples of the obstacles (rectangular boxes) that the robot can pass beneath. The capability of passing through a confined space can be predicted from the envelope contour of the crawling robot with different voltage applied. (fig. S6) More specifically, we set up a confined tunnel with a height of only 3 mm and length of 30 mm. Note that with no constraint, the maximum height of the crawling robot can reach up to 8.9 mm in forward mode and 14.5 mm in reverse mode. Figure 6D shows the snapshots of the robot passing through this confined tunnel and passing back to return to the initial location. When the robot passes through, the actuators are operated just as described above and shown in Fig. 6A. When the robot retrieves, actuator B changes from reverse mode to forward mode so that the robot gets pushed back into the tunnel. Then, actuator A is turned on in reverse mode, which drives the body parts free of the geometrical constraint without changing the moving direction. We note that the multi-gait capability distinct our soft robot from the most reported soft crawlers under different actuations, where they are capable of neither changing their gaits to go through confined spaces nor moving bidirectionally (*2*, *12*, *53*, *54*). This capability of passing through small confined space in forward and backward locomotion can be of promising potential for many applications such as search and rescue.

## DISCUSSION

Although crawling robots have been studied intensively in recent decades, most of the reported crawling robots only showed simple bending motion with constant curvature. Only a few works demonstrated bidirectional motion. For example, Lu *et al.* (*55*) reported a bidirectional peristaltic crawling robot by sequential control of each segment. However, this crawling robot requires high voltage (6 kV) and sophisticated controls (collaborative control of four segments). In addition, this robot is composed of rigid skeletons instead of completely soft materials. Xiao *et al.* (*56*) developed a vibration-driven biomimetic soft robot, where the two actuators are excited at different frequencies (1 and 50 Hz, respectively). By switching the actuation frequency of the two actuators, the robot can move bidirectionally. However, the voltage required is still very high (800 V). Wu *et al.* (*57*) showed a magnetic field–driven actuator, which can switch crawling direction by changing the magnetic field. However, a magnetic field control is generally more complicated compared with electrothermal control. Table S1 summarizes soft robots based on electrothermal actuation. In particular, Wang *et al.* (*39*) showed bidirectional crawler consisting of two heaters, one causing upward bending and the other downward bending. Each locomotion, either forward or reverse, involves two heaters and four sequential steps to complete one stride (one cycle of crawling locomotion); the two heaters must be powered on and off following a coordinated sequence. Note that the upward/downward bending requires different stack orders of materials to construct the thermal bimorph, which makes the device three layers instead of two, increasing the complexity of fabrication.

For comparison, our work adopts distributed heating to control the local curvature of the crawling robot. The designed body profiles are inspired by the curvature distribution of a caterpillar, which can self-regulate the friction with the ground, key to achieving the two-directional crawling. For each locomotion, only one actuator and two steps (simply power on and off of the actuator) are required for one cycle of crawling locomotion. In addition, as indicated in a first-order analysis (in section S8), the energy efficiency of our crawling robot is much higher than that reported in (*39*) using the same actuation mechanism (electrothermal) and same material (LCE). In short, the main features of our bioinspired soft crawler compared with other two-directional crawlers include complete softness (no rigid parts), low voltage (less than 5 V), simple fabrication and control (square-wave input on one actuator at a time), dynamic body profile control (capability of passing through confined gaps with limited space), relatively fast speed, and higher energy efficiency (for electrothermal actuation).

To sum up, we designed and fabricated a caterpillar-inspired, energy-efficient crawling robot with bidirectional locomotion, enabled by joule heating of a patterned soft heater consisting of AgNW networks in an LCE-based thermal bimorph actuator. The different actuation modes are controlled by joule heating of designed AgNW heating patterns. With designed heating patterns and programmable heating, different temperature and curvature distribution can be achieved, resulting in different frictions between the front and rear ends with the ground. FEA was conducted to model the friction mechanism of the locomotion modes, which agreed very well with the experimental results. The locomotion speed of the two crawling modes (forward and reverse) as a function of the applied current and frequency was characterized. To demonstrate the crawling robot for potential applications, we tested with a scenario of passing through a confined gap with limited space. The strategy of distributed and programmable heating with thermal responsive materials offers exciting capabilities for smart and multifunctional soft robots.

## MATERIALS AND METHODS

### LCE ribbon fabrication

The LCE samples were synthesized by modifying previously reported thiol-acrylate Michael addition reaction method. The liquid crystal mesogenic monomer, 1,4-bis-[4-(3-acryloyloxypropyloxy)benzoyloxy]-2-methylbenzene (RM 257), was purchased from Wilshire Technologies and used without further modification. In a typical synthesis process, 2 g of RM 257 was first fully dissolved in 0.7 g of toluene at 85°C with magnetic stirring, followed by cooling down to room temperature. Then, 0.42 g of the chain extender 2,2′-(ethylenedioxy) diethanethiol (Sigma-Aldrich), 0.18 g of crosslinker pentaerythritol tetrakis (3-mercaptopropionate) (Sigma-Aldrich), and 0.012 g of the photoinitiator (2-hydroxyethoxy)-2-methylpropiophenone (Sigma-Aldrich) were added in the solution. The solution was then well dissolved at 85°C and cooled down to room temperature again. Subsequently, 0.288 g of the dipropyl amine (DPA, Sigma-Aldrich) solution (2 weight %, in toluene) was added in the solution that serves as the catalyst. After being fully mixed and degassed, the solution was carefully poured in the prepared mold (9 cm in length, 3 mm in width, and 1 mm in depth). Next, the mold was placed in a closed container overnight for fully reaction. A first-cured LCE sample can be obtained after dried at 80°C for 1 day. When the LCE ribbon was fully dried, it was uniaxially stretched to 100% strain, followed by exposure to 365-nm ultraviolet irradiation at an intensity of 20 mJ/cm^2^ for 10 min.

### Synthesis of AgNWs

First, 60 ml of a 0.147 M polyvinyl pyrrolidone (PVP) (molecular weight of ~40,000; Sigma-Aldrich) solution in ethylene glycol (EG) was added to a flask, to which a stir bar was added; the solution was then suspended in an oil bath (temperature of 151.5°C) and heated for 1 hour under magnetic stirring (150 rpm). Then, 200 μl of a 24 M CuCl_2_ (CuCl_2_·2H_2_O, 99.999+%; Sigma-Aldrich) solution in EG was injected into the PVP solution. The mixture solution was then injected with 60 ml of a 0.094 M AgNO_3_ (99+%; Sigma-Aldrich) solution in EG (*58*).

### Fabrication of the crawling robot

A patterned mask was made by laser cutting a thin polyimide (PI) film on top of a glass slide. Then, the prepared AgNW solution was drop-casted on the masked glass slide, which was then placed onto a hot plate at 50°C to evaporate the solvent. After the solvent was evaporated, the PI mask was removed together with AgNWs on top. Liquid PDMS (SYLGARD 184, Dow Inc.) with a weight ratio of 10:1 was mixed thoroughly with CB (weight ratio between liquid PDMS and CB was 4:1 and then dropped on top of the patterned AgNWs on glass slide). After spin coating, the PDMS/CB layer was controlled with uniform thickness. The AgNW/PDMS/CB composite was cured at 70°C for 1 hour (*13*, *49*). Then, the PDMS/CB side of the composite film and the surface of prepared LCE ribbon were plasma-treated for 20 s and then laminated together with pressure to form a strong bonding. The Cu wires were attached to the eight ends of the conductive patterns by silver epoxy (MG Chemicals).

### FEA of the two crawling modes

The crawler is modeled as a bilayer 3D deformable part, while the ground is modeled as a rigid part. The geometry of all actuators was taken from experimental data and imported into Abaqus CAE and then meshed with solid quadratic tetrahedral elements (C3D10H). A mesh refinement study was applied to verify the accuracy of the mesh. The thermal expansion of the LCE elastomer is set to be orthotropic according to the prestretch direction in experiments, where the expansion coefficients are −0.1, 0, and 0 in the *x*, *y*, and *z* directions, respectively. An equal friction coefficient (0.3) is applied between the bottom and side surface of the crawler and the substrate to simulate the varying friction force induced by the morphology with a predefined temperature field applied during dynamic explicit analysis. The thermal expansion rate of the LCE elastomer is set to be orthotropic according to the prestretched direction in the experiment. The Young’s modulus of the LCE was taken from tensile experiment shown in fig. S4. The Young’s modulus of AgNW/PDMS is taken from Dow Inc. datasheet. The effect of AgNWs on the composite was neglected because of the minimal thickness ratio (<1:20).

## Supplementary Materials

### This PDF file includes:

Sections S1 to S8 Figs. S1 to S6 Table S1 Legends for movies S1 to S3 References
