# Supplementary material for: Caterpillar-inspired soft crawling robot with distributed programmable thermal actuation
Source: Sci Adv. 2023 Mar 22;9(12):eadf8014. doi: 10.1126/sciadv.adf8014 (PMC10032605; doi:10.1126/sciadv.adf8014)
Supplement: Supplementary file 1 — Sections S1 to S8 Figs. S1 to S6 Table S1 Legends for movies S1 to S3 References [file sciadv.adf8014_sm.pdf]

Science Advances
AAAS

Supplementary Materials for

Caterpillar-inspired soft crawling robot with distributed programmable thermal actuation

Shuang Wu *et al.*

Corresponding author: Yong Zhu, yzhu7@ncsu.edu

Sci. Adv. **9**, eadf8014 (2023)
DOI: 10.1126/sciadv.adf8014

The PDF file includes:

Sections S1 to S8
Figs. S1 to S6
Table S1
Legends for movies S1 to S3
References

Other Supplementary Material for this manuscript includes the following:

Movies S1 to S3

1. SEM image of AgNW network.

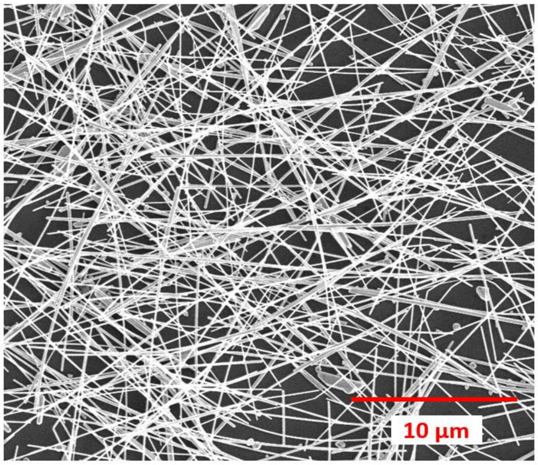

Fig. S1 SEM image of dropcasted AgNW network on silicon wafer.

2. Thermal and mechanical measurement of PDMS and PDMS/CB.

The inset figure in Fig. S2A shows the experimental setup for measuring the thermal conductivity of PDMS/CB. The same AgNW/PDMS heater is attached on top of two cuboid test samples (a PDMS sample and a PDMS/CB sample with identical size and shape). A copper heat sink is connected to the bottom of the samples. According to the equation of thermal conductivity :

$$k = \frac{qt}{A\Delta T} \tag{S-1}$$

where ΔT is the temperature difference along the heat flux direction, t and A are the thickness and cross section area of the sample, q is the heat transfer in the sample.

With the same input power for joule heating, identical shape and size, and with the heat dissipation to ambient air neglected, we assume the same heat transfer in the PDMS and PDMS/CB samples. Basing on equation (1):

$$k_{PDMS/CB} = \frac{\Delta T_{PDMS}}{\Delta T_{PDMS/CB}} k_{PDMS} \tag{S-2}$$

where k_{PDMS}= 0.16 W/mK (from the data sheet of Dow Inc). Based on the temperature difference captured in the IR images, the thermal conductivity $k_{PDMS/CB}$= 0.21 W/mK.

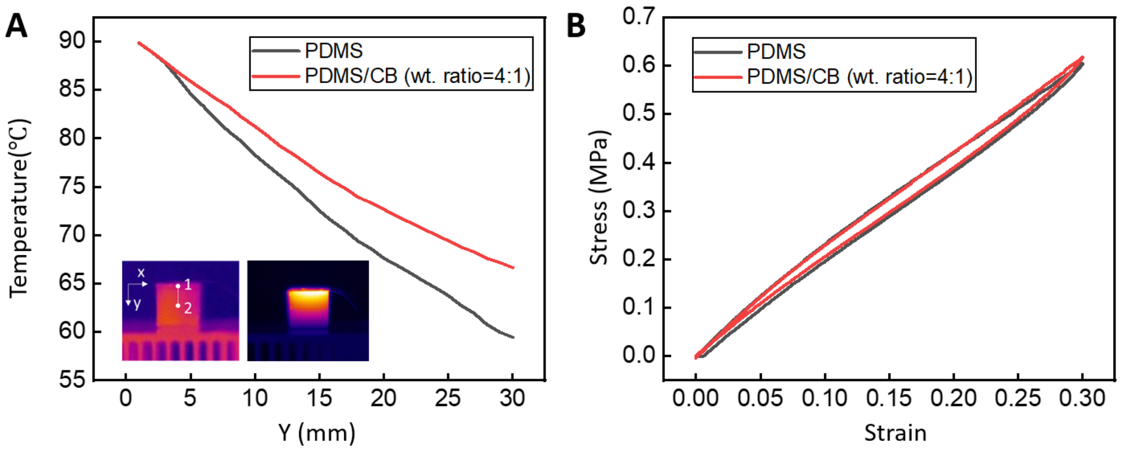

Fig. S2 Thermal and mechanical effect of doping CB in PDMS. (**A**) Thermal conductivity test of PDMS and PDMS/CB (with weight ratio of 4:1). (**B**) Stress strain curve of PDMS and PDMS/CB (with weight ratio of 4:1).

3. Temperature gradient of the AgNW/PDMS/CB actuator

The IR images on the top and bottom of the actuator reveal an average temperature difference of 0.3 °C in the steady state of the joule heating (after 3 s). We also estimated the temperature difference theoretically based on the experimentally measured parameters.

According to the equation of thermal conduction of a two-layer composite, temperature difference between the top PDMS/CB layer and bottom LCE layer is given by,

$$\Delta T = \frac{q}{A}\left(\frac{t_{PDMS/CB}}{k_{PDMS/CB}} + \frac{t_{LCE}}{k_{LCE}}\right) \tag{S-3}$$

where q is the heat transfer experimentally measured using the same AgNW/PDMS/CB heater on a short cuboid of PDMS/CB (q=0.032 W) (using the setup shown in the inset of Fig. S2), $t_{PDMS/CB}$, t_{LCE} and $k_{PDMS/CB}$, k_{LCE} are the thickness and thermal conductivity of the two layers, respectively ($t_{PDMS/CB}$=0.06 mm, t_{LCE}=0.251 mm, $k_{PDMS/CB}$=0.21 W/mK, k_{LCE}=0.22 W/mK (59)), and A is the cross section area of the heat flux (for both layers A=1.6 cm^2). Based on all these parameters, the estimated temperature difference ΔT=0.287 °C, very close to experimental result using the IR images.

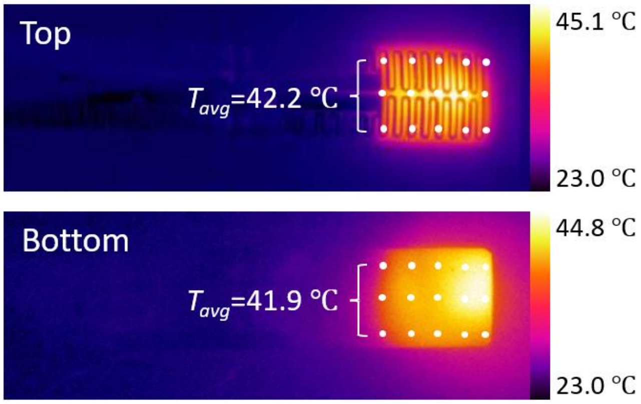

Fig. S3 IR images of the top and bottom surface of the actuator. The comparison shows an average temperature difference of 0.3 °C (sampled from 15 points in the heating area).

4. Stress-strain curve of LCE

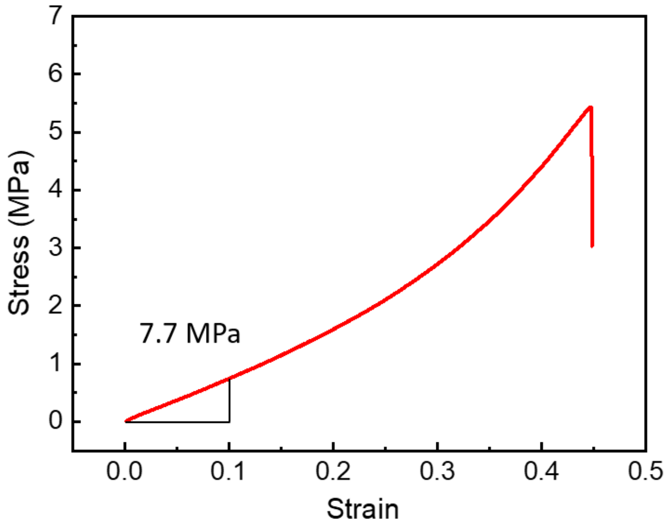

Fig. S4 Stress-strain curve of the LCE ribbon.

5. Strain-temperature relationship of LCE

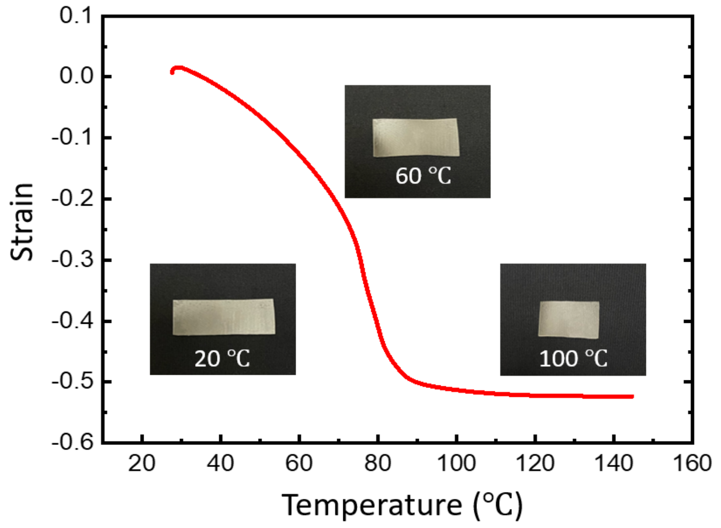

Fig. S5 Strain-temperature relationship of the fabricated LCE ribbon. The inset figures show the LCE sample in an oven at different temperatures.

6. Profile control of the crawling motion

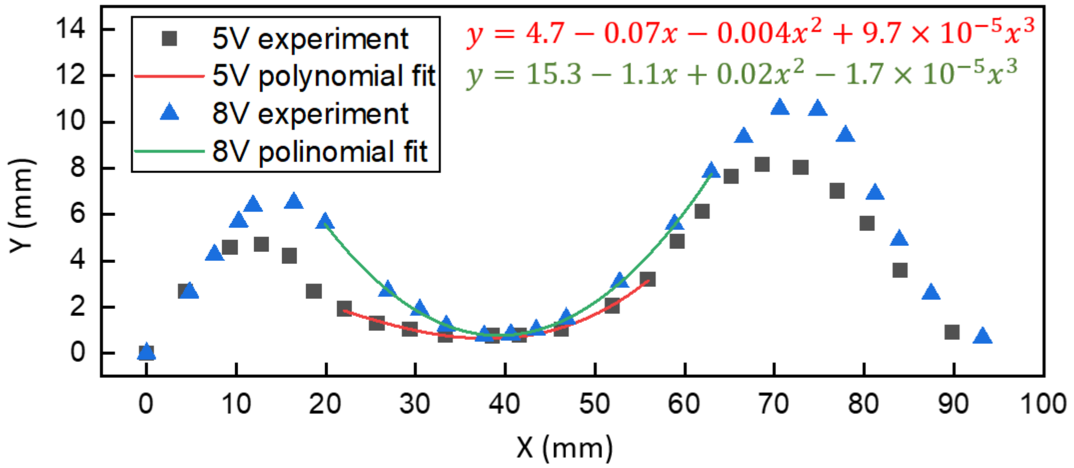

Fig. S6 The crawling robot body profile control. The body profile contour and corresponding polynomial fit of the crawling motion with 5V and 8V applied.

7. Summary of the soft robots based on electrothermal actuation

Table S1 Summary of the soft robots based on electrothermal actuation

	Thermal responsive materials	Heater	Distributed heater	Heater programmability	Locomotion performance
(41)	LCE/PI	Ni-Cr wires	×	×	Crawling (1-directional)
(39)	LCE/PI	Cr-Au mesh	√	√	Crawling (2-directional)
(60)	LCE/PDMS	AgNWs	×	×	No crawling demonstrated
(61)	LCE/Silicone	Liquid metal	×	×	No crawling demonstrated
(14)	LCE	Cu wires	√	√	Walking (multi-gait)
(62)	GO	Conductive fabric	×	×	Crawling (1-directional)
(63)	LCE	CNT	×	×	Crawling (1-directional) & jumping
(64)	Nylon/CNT	CNT	×	×	Crawling (1-directional)
(65)	SMA	SMA	×	×	Crawling (1-directional)
Our work	LCE/PDMS	AgNWs	√	√	Crawling (2-directional & bioinspired body profile control)

8. Estimate of the energy efficiency

Here we present a first-order estimate of the energy efficiency of our soft robot. The energy efficiency depends on the actuation mechanism and materials involved, so here we only conduct a preliminary analysis of our soft robot and limit the comparison to a specific study reporting bidirectional locomotion with the same actuation mechanism (electrothermal) and the same material (LCE) (39).

In our work, the input power are 0.109 W and 0.174 W for each locomotion (0.5 mm/s for forward locomotion and 0.72 mm/s for reverse locomotion), under the current of 30 mA. Cost of transport CoT=$E/(mgd)$, as defined in literature (66), is used to characterize the energy efficiency, where the E is the input energy, m is the mass, g is gravitational constant, and d is moved distance. In our work, the mass m is 0.46 g, so CoT=4.88×10^4 for forward locomotion and CoT=5.36×10^4 for reverse motion.

In (39), the power is 0.069 W for each locomotion (0.032 mm/s for forward locomotion and 0.021 mm/s for reverse locomotion) and the mass m is 0.29 g. CoT=7.36×10^5 for forward locomotion and CoT=1.07×10^6 for reverse motion.

It can be seen that CoT in our work is approximately 0.05 and 0.07 times of the soft robot in (39) in forward and reverse locomotion, respectively, therefore the energy efficiency of our crawling robot is much higher for the same actuation mechanism and material.

Other Supplementary Materials:

Movie S1
Forward mode locomotion of the crawling robot.

Movie S2
Reverse mode locomotion of the crawling robot.

Movie S3
The crawling robot passing through a confined tunnel and passing back to return to the initial location.

REFERENCES AND NOTES

1. C. Laschi, M. Cianchetti, B. Mazzolai, L. Margheri, M. Follador, P. Dario, Soft robot arm inspired by the octopus. *Adv. Robot.* **26**, 709–727 (2012).

2. R. F. Shepherd, F. Ilievski, W. Choi, S. A. Morin, A. A. Stokes, A. D. Mazzeo, X. Chen, M. Wang, G. M. Whitesides, Multigait soft robot. *Proc. Natl. Acad. Sci. U.S.A* **108**, 20400–20403 (2011).

3. T. Li, G. Li, Y. Liang, T. Cheng, J. Dai, X. Yang, B. Liu, Z. Zeng, Z. Huang, Y. Luo, T. Xie, W. Yang, Fast-moving soft electronic fish. *Sci. Adv.* **3**, e1602045 (2017).

4. A. D. Marchese, C. D. Onal, D. Rus, Autonomous soft robotic fish capable of escape maneuvers using fluidic elastomer actuators. *Soft Robot.* **1**, 75–87 (2014).

5. Y. Cao, Y. Liu, Y. Chen, L. Zhu, Y. Yan, X. Chen, A novel slithering locomotion mechanism for a snake-like soft robot. *J. Mech. Phys. Solids* **99**, 304–320 (2017).

6. C. D. Onal, D. Rus, Autonomous undulatory serpentine locomotion utilizing body dynamics of a fluidic soft robot. *Bioinspir. Biomim.* **8**, 026003 (2013).

7. B. Zhang, Y. Fan, P. Yang, T. Cao, H. Liao, Worm-like soft robot for complicated tubular environments. *Soft Robot.* **6**, 399–413 (2019).

8. H. Niu, R. Feng, Y. Xie, B. Jiang, Y. Sheng, Y. Yu, H. Baoyin, X. Zeng, Magworm: A biomimetic magnet embedded worm-like soft robot. *Soft Robot.* **8**, 507–518 (2021).

9. L. Sun, Z. Chen, F. Bian, Y. Zhao, Bioinspired soft robotic caterpillar with cardiomyocyte drivers. *Adv. Funct. Mater.* **30**, 1907820 (2020).

10. H.-T. Lin, G. G. Leisk, B. Trimmer, GoQBot: A caterpillar-inspired soft-bodied rolling robot. *Bioinspir. Biomim.* **6**, 026007 (2011).

11. M. T. Tolley, R. F. Shepherd, B. Mosadegh, K. C. Galloway, M. Wehner, M. Karpelson, R. J. Wood, G. M. Whitesides, A resilient, untethered soft robot. *Soft Robot.* **1**, 213–223 (2014).

12. Y. Tang, Y. Chi, J. Sun, T.-H. Huang, O. H. Maghsoudi, A. Spence, J. Zhao, H. Su, J. Yin, Leveraging elastic instabilities for amplified performance: Spine-inspired high-speed and high-force soft robots. *Sci. Adv.* **6**, eaaz6912 (2020).

13. S. Yao, J. Cui, Z. Cui, Y. Zhu, Soft electrothermal actuators using silver nanowire heaters. *Nanoscale* **9**, 3797–3805 (2017).

14. Q. He, Z. Wang, Y. Wang, A. Minori, M. T. Tolley, S. Cai, Electrically controlled liquid crystal elastomer–based soft tubular actuator with multimodal actuation. *Sci. Adv.* **5**, eaax5746 (2019).

15. Y. Zhao, Y. Chi, Y. Hong, Y. Li, S. Yang, J. Yin, Twisting for soft intelligent autonomous robot in unstructured environments. *Proc. Natl. Acad. Sci. U.S.A.* **119**, e2200265119 (2022).

16. Y. Li, Y. Teixeira, G. Parlato, J. Grace, F. Wang, B. D. Huey, X. Wang, Three-dimensional thermochromic liquid crystal elastomer structures with reversible shape-morphing and color-changing capabilities for soft robotics. *Soft Matter* **18**, 6857–6867 (2022).

17. S. Wu, G. L. Baker, J. Yin, Y. Zhu, Fast thermal actuators for soft robotics. *Soft Robot.* **9**, 1031–1039 (2022).

18. E. Acome, S. Mitchell, T. Morrissey, M. Emmett, C. Benjamin, M. King, M. Radakovitz, C. Keplinger, Hydraulically amplified self-healing electrostatic actuators with muscle-like performance. *Science* **359**, 61–65 (2018).

19. J. Shintake, V. Cacucciolo, D. Floreano, H. Shea, Soft robotic grippers. *Adv. Mater.* **30**, 1707035 (2018).

20. W. Hu, G. Z. Lum, M. Mastrangeli, M. Sitti, Small-scale soft-bodied robot with multimodal locomotion. *Nature* **554**, 81–85 (2018).

21. G. Mao, M. Drack, M. Karami-Mosammam, D. Wirthl, T. Stockinger, R. Schwödiauer, M. Kaltenbrunner, Soft electromagnetic actuators. *Sci. Adv.* **6**, eabc0251 (2020).

22. Y. Kim, H. Yuk, R. Zhao, S. A. Chester, X. Zhao, Printing ferromagnetic domains for untethered fast-transforming soft materials. *Nature* **558**, 274–279 (2018).

23. J. C. Nawroth, H. Lee, A. W. Feinberg, C. M. Ripplinger, M. L. McCain, A. Grosberg, J. O. Dabiri, K. K. Parker, A tissue-engineered jellyfish with biomimetic propulsion. *Nat. Biotechnol.* **30**, 792–797 (2012).

24. I. Must, E. Sinibaldi, B. Mazzolai, A variable-stiffness tendril-like soft robot based on reversible osmotic actuation. *Nat. Commun.* **10**, 344 (2019).

25. Y. Bar-Cohen, Q. Zhang, Electroactive polymer actuators and sensors. *MRS bull.* **33**, 173–181 (2008).

26. L. Chen, C. Liu, K. Liu, C. Meng, C. Hu, J. Wang, S. Fan, High-performance, low-voltage, and easy-operable bending actuator based on aligned carbon nanotube/polymer composites. *ACS Nano* **5**, 1588–1593 (2011).

27. H. Kim, H. Lee, I. Ha, J. Jung, P. Won, H. Cho, J. Yeo, S. Hong, S. Han, J. Kwon, K.-J. Cho, S. H. Ko, Biomimetic color changing anisotropic soft actuators with integrated metal nanowire percolation network transparent heaters for soft robotics. *Adv. Funct. Mater.* **28**, 1801847 (2018).

28. E. Hawkes, B. An, N. M. Benbernou, H. Tanaka, S. Kim, E. D. Demaine, D. Rus, R. J. Wood, Programmable matter by folding. *Proc. Natl. Acad. Sci. U.S.A.* **107**, 12441–12445 (2010).

29. S. Yao, J. Yang, F. R. Poblete, X. Hu, Y. Zhu, Multifunctional Electronic Textiles Using Silver Nanowire Composites. *ACS Appl. Mater. Interfaces* **11**, 31028–31037 (2019).

30. J. W. Lee, R. Xu, S. Lee, K.-I. Jang, Y. Yang, A. Banks, K. J. Yu, J. Kim, S. Xu, S. Ma, S. W. Jang, P. Won, Y. Li, B. H. Kim, J. Y. Choe, S. Huh, Y. H. Kwon, Y. Huang, U. Paik, J. A. Rogers, Soft, thin skin-mounted power management systems and their use in wireless thermography. *Proc. Natl. Acad. Sci. U.S.A.* **113**, 6131–6136 (2016).

31. X. Qian, Q. Chen, Y. Yang, Y. Xu, Z. Li, Z. Wang, Y. Wu, Y. Wei, Y. Ji, Untethered recyclable tubular actuators with versatile locomotion for soft continuum robots. *Adv. Mater.* **30**, 1801103 (2018).

32. A. Kotikian, C. McMahan, E. C. Davidson, J. M. Muhammad, R. D. Weeks, C. Daraio, J. A. Lewis, Untethered soft robotic matter with passive control of shape morphing and propulsion. *Sci. Robot.* **4**, eaax7044 (2019).

33. S. Palagi, A. G. Mark, S. Y. Reigh, K. Melde, T. Qiu, H. Zeng, C. Parmeggiani, D. Martella, A. Sanchez-Castillo, N. Kapernaum, G. Frank, D. S. Wiersma, E. Lauga, P. Fischer, Structured light enables biomimetic swimming and versatile locomotion of photoresponsive soft microrobots. *Nat. Mater.* **15**, 647–653 (2016).

34. H. Tian, Z. Wang, Y. Chen, J. Shao, T. Gao, S. Cai, Polydopamine-coated main-chain liquid crystal elastomer as optically driven artificial muscle. *ACS Appl. Mater. Interfaces* **10**, 8307–8316 (2018).

35. M. Camacho-Lopez, H. Finkelmann, P. Palffy-Muhoray, M. Shelley, Fast liquid-crystal elastomer swims into the dark. *Nat. Mater.* **3**, 307–310 (2004).

36. Y. Wang, A. Dang, Z. Zhang, R. Yin, Y. Gao, L. Feng, S. Yang, Repeatable and reprogrammable shape morphing from photoresponsive gold nanorod/liquid crystal elastomers. *Adv. Mater.* **32**, 2004270 (2020).

37. S. Schuhladen, F. Preller, R. Rix, S. Petsch, R. Zentel, H. Zappe, Iris-like tunable aperture employing liquid-crystal elastomers. *Adv. Mater.* **26**, 7247–7251 (2014).

38. J. Sun, Y. Wang, W. Liao, Z. Yang, Ultrafast, High-Contractile Electrothermal-Driven Liquid Crystal Elastomer Fibers towards Artificial Muscles. *Small* **17**, 2103700 (2021).

39. C. Wang, K. Sim, J. Chen, H. Kim, Z. Rao, Y. Li, W. Chen, J. Song, R. Verduzco, C. Yu, Soft ultrathin electronics innervated adaptive fully soft robots. *Adv. Mater.* **30**, 1706695 (2018).

40. H. Liu, H. Tian, J. Shao, Z. Wang, X. Li, C. Wang, X. Chen, An electrically actuated soft artificial muscle based on a high-performance flexible electrothermal film and liquid-crystal elastomer. *ACS Appl. Mater. Interfaces* **12**, 56338–56349 (2020).

41. Y.-Y. Xiao, Z.-C. Jiang, X. Tong, Y. Zhao, Biomimetic locomotion of electrically powered "Janus" soft robots using a liquid crystal polymer. *Adv. Mater.* **31**, 1903452 (2019).

42. J. Brackenbury, Caterpillar kinematics. *Nature* **390**, 453 (1997).

43. J. Brackenbury, Novel locomotory mechanisms in caterpillars: Life-line climbing in Epinotia abbreviana (Tortricidae) and Yponomeuta padella (Yponomeutidae). *Physiol. Entomol.* **21**, 7–14 (1996).

44. S. Yao, Y. Zhu, Nanomaterial-enabled stretchable conductors: Strategies, materials and devices, *Adv. Mater.* **27**, 1480–1511 (2015).

45. S. Hong, H. Lee, J. Lee, J. Kwon, S. Han, Y. D. Suh, H. Cho, J. Shin, J. Yeo, S. H. Ko, Highly stretchable and transparent metal nanowire heater for wearable electronics applications. *Adv. Mater.* **27**, 4744–4751 (2015).

46. S. Choi, J. Park, W. Hyun, J. Kim, J. Kim, Y. B. Lee, C. Song, H. J. Hwang, J. H. Kim, T. Hyeon, D.-H. Kim, Stretchable heater using ligand-exchanged silver nanowire nanocomposite for wearable articular thermotherapy. *ACS Nano* **9**, 6626–6633 (2015).

47. F. Xu, Y. Zhu, Highly conductive and stretchable silver nanowire conductors. *Adv. Mater.* **24**, 5117–5122 (2012).

48. L. Song, A. C. Myers, J. J. Adams, Y. Zhu, Stretchable and reversibly deformable radio frequency antennas based on silver nanowires. *ACS Appl. Mater. Interfaces* **6**, 4248–4253 (2014).

49. S. Yao, Y. Zhu, Wearable multifunctional sensors using printed stretchable conductors made of silver nanowires. *Nanoscale* **6**, 2345–2352 (2014).

50. C. M. Yakacki, M. Saed, D. P. Nair, T. Gong, S. M. Reed, C. N. Bowman, Tailorable and programmable liquid-crystalline elastomers using a two-stage thiol–acrylate reaction. *RSC Adv.* **5**, 18997–19001 (2015).

51. M. O. Saed, A. H. Torbati, D. P. Nair, C. M. Yakacki, Synthesis of programmable main-chain liquid-crystalline elastomers using a two-stage thiol-acrylate reaction. *J. Vis. Exp.* **107**, e53546 (2016).

52. S. Timoshenko, Analysis of bi-metal thermostats. *J. Opt. Soc. Am.* **11**, 233–255 (1925).

53. A. Rafsanjani, Y. Zhang, B. Liu, S. M. Rubinstein, K. Bertoldi, Kirigami skins make a simple soft actuator crawl. *Sci. Robot.* **3**, eaar7555 (2018).

54. Y. Chi, Y. Tang, H. Liu, J. Yin, Leveraging monostable and bistable pre-curved bilayer actuators for high-performance multitask soft robots. *Adv. Mater.Technol.* **5**, 2000370 (2020).

55. X. Lu, K. Wang, T. Hu, Development of an annelid-like peristaltic crawling soft robot using dielectric elastomer actuators. *Bioinspir. Biomim.* **15**, 046012 (2020).

56. Y. Xiao, J. Mao, Y. Shan, T. Yang, Z. Chen, F. Zhou, J. He, Y. Shen, J. Zhao, T. Li, Y. Lou, Anisotropic electroactive elastomer for highly maneuverable soft robotics. *Nanoscale* **12**, 7514–7521 (2020).

57. S. Wu, Q. Ze, R. Zhang, N. Hu, Y. Cheng, F. Yang, R. Zhao, Symmetry-breaking actuation mechanism for soft robotics and active metamaterials. *ACS Appl. Mater. Interfaces* **11**, 41649–41658 (2019).

58. Y. Sun, B. Gates, B. Mayers, Y. Xia, Crystalline silver nanowires by soft solution processing. *Nano Lett.* **2**, 165–168 (2002).

59. J. Shin, M. Kang, T. Tsai, C. Leal, P. V. Braun, D. G. Cahill, Thermally functional liquid crystal networks by magnetic field driven molecular orientation. *ACS Macro Lett.* **5**, 955–960 (2016).

60. J. Liu, L. Xu, C. He, X. Lu, F. Wang, Transparent low-voltage-driven soft actuators with silver nanowires Joule heaters. *Polym. Chem.* **12**, 5251–5256 (2021).

61. B. Ma, C. Xu, L. Cui, C. Zhao, H. Liu, Magnetic printing of liquid metal for perceptive soft actuators with embodied intelligence. *ACS Appl. Mater. Interfaces* **13**, 5574–5582 (2021).

62. R. Wang, L. Han, C. Wu, Y. Dong, X. Zhao, Localizable, identifiable, and perceptive untethered light-driven soft crawling robot. *ACS Appl. Mater. Interfaces* **14**, 6138–6147 (2022).

63. C. Ahn, X. Liang, S. Cai, Bioinspired design of light-powered crawling, squeezing, and jumping untethered soft robot. *Adv. Mater.Technol.* **4**, 1900185 (2019).

64. Z. Liu, R. Zhang, Y. Xiao, J. Li, W. Chang, D. Qian, Z. Liu, Somatosensitive film soft crawling robots driven by artificial muscle for load carrying and multi-terrain locomotion. *Mater. Horiz.* **8**, 1783–1794 (2021).

65. T. Umedachi, V. Vikas, B. A. Trimmer, Highly deformable 3-D printed soft robot generating inching and crawling locomotions with variable friction legs, in *Proceedings of the 2013 IEEE/RSJ International Conference on Intelligent Robots and Systems* (IEEE, 2013), pp. 4590–4595.

66. S. Collins, A. Ruina, R. Tedrake, M. Wisse, Efficient bipedal robots based on passive-dynamic walkers. *Science* **307**, 1082–1085 (2005).
